# Threshold‐dependent association between non‐rapid eye movement obstructive sleep apnea and interictal epileptiform discharges: A hospital study

**DOI:** 10.1111/jsr.14385

**Published:** 2024-10-23

**Authors:** Meina Wu, Pei Xue, Jinzhu Yan, Christian Benedict

**Affiliations:** ^1^ Department of Neurology and Sleep Medical Center Fujian Provincial Governmental Hospital Fuzhou China; ^2^ Department of Pharmaceutical Biosciences Uppsala University Uppsala Sweden

**Keywords:** epilepsy, interictal epileptiform discharges, obstructive sleep apnea, sleep

## Abstract

Obstructive sleep apnea frequently coexists with epilepsy, potentially influencing its pathophysiology. However, the effect of obstructive sleep apnea severity on interictal epileptiform discharges is not well understood. To explore this, we studied 108 Asian patients with epilepsy who underwent single‐night polysomnography. We utilized generalized linear models, adjusting for age, sex, epilepsy type (focal versus generalized), antiepileptic medication use and disease duration, to analyse the relationship between obstructive sleep apnea severity, as measured by the apnea–hypopnea index, and interictal epileptiform discharge frequency during non‐rapid eye movement and rapid eye movement sleep. Our analysis revealed that severe obstructive sleep apnea (apnea–hypopnea index ≥ 30) was associated with a higher frequency of interictal epileptiform discharges during non‐rapid eye movement sleep (*p* = 0.04), but no such association was observed during rapid eye movement sleep. Additionally, the frequency of interictal epileptiform discharges in non‐rapid eye movement sleep was positively correlated with the wake time between sleep onset and offset (*p* = 0.03). Further studies are warranted to validate our findings across diverse ethnicities, and over multiple nights of sleep and interictal epileptiform discharge recordings.

## 1 INTRODUCTION

A meta‐analysis found that ~33.4% of patients with epilepsy experience mild‐to‐severe obstructive sleep apnea (OSA; Lin et al., 2017), a condition characterized by at least five episodes of partial or complete upper airway obstruction per hour of sleep (Berry et al., 2017). Notably, patients with epilepsy have a higher prevalence of interictal epileptiform discharges (IEDs) during non‐rapid eye movement (NREM) sleep compared with rapid eye movement (REM) sleep (Malow et al., 1997). IEDs are characterized by spikes, sharp waves or other epileptiform patterns observed on electroencephalogram (EEG) recordings, indicating a predisposition to epilepsy without necessarily resulting in a seizure (Kural et al., 2020).

Although treating OSA in patients with epilepsy has been shown to reduce the frequency of IEDs (Pornsriniyom et al., 2014), suggesting that mitigating disordered breathing during sleep may help control epilepsy, the specific effects of OSA events during different sleep stages on IEDs are still not fully understood. To investigate this, our study involved 108 patients with epilepsy with varying degrees of OSA severity who underwent a single night of simultaneous sleep and IEDs recording.

## 2 METHODS

### 2.1 Participants and procedure

This hospital‐based study, conducted in accordance with the STROBE reporting guidelines, was carried out at the Neurology and Sleep Medicine Center of Fujian Provincial Governmental Hospital in China, from 1 July 2018 to 31 December 2022. It received approval from the Ethics Committee of Fujian Provincial Governmental Hospital and adhered to the principles outlined in the Declaration of Helsinki. All participants provided written informed consent, specifying that their data could be used for scientific publications.

A total of 119 Asian patients with epilepsy, seeking treatment for their clinical symptoms at neurology and sleep medicine centres, were enrolled in the study due to concerns about possible OSA. Clinical data, including neurological examination results, seizure semiology, EEG, neuroimaging findings and medication usage, were documented. Out of the 119 patients, 108 met the inclusion criteria, which were a minimum of 4 hr of sleep to ensure a valid assessment of overall sleep and sleep stage‐specific OSA; and a confirmed diagnosis of epilepsy. Confirmation of epilepsy was achieved through prior neurological assessments documented in patient records or, if newly diagnosed, according to the 2017 International Anti‐Epilepsy Alliance criteria, which included EEG confirmation of ED (Scheffer et al., 2017). Exclusion criteria included the presence of concurrent neurological or psychiatric conditions known to impact sleep, such as narcolepsy, anxiety disorders and depression; epilepsy induced by antibiotic drugs or associated with specific epilepsy syndromes; severe systemic illnesses (e.g. cancer); and inability to cooperate with the examination protocol. Four initially enrolled patients were subsequently excluded due to seizure occurrence during the in‐hospital night, and an additional seven patients were excluded as their sleep duration was less than 4 hr.

### 2.2 One‐night sleep assessment

Participants underwent overnight polysomnography with video‐EEG monitoring for a minimum of 7 hr. We utilized a comprehensive diagnostic sleep system (Alice 6 LDxS, Philips Respironics, USA), and included parameters such as EEG, electrooculography, electromyography, oral airflow, rib cage and abdominal movements, and blood oxygen saturation. Monitored parameters encompassed total sleep time, sleep‐onset latency, wake time after sleep onset, sleep efficiency, NREM sleep, REM sleep and apnea–hypopnea index (AHI), analysed according to the American Academy of Sleep Medicine guidelines (Berry et al., 2017).

### 2.3 IED assessment

The IEDs were visually identified by three experienced raters, all of whom work clinically at the Department of Neurology and Sleep Medicine at Fujian Provincial Governmental Hospital in Fuzhou, China. IEDs were counted only when a consensus was reached. To be considered, IED had to meet at least four of the following six criteria (Kural et al., 2020): di‐ or tri‐phasic waves with sharp or spiky morphology; distinct wave duration compared with ongoing background activity; asymmetry of waveform: sharply rising ascending phase and gradual decaying descending phase, or vice versa; transient followed by an associated slow after‐wave; presence of IEDs disrupts surrounding background activity; and distribution of negative and positive potentials on the scalp suggests a brain source, best assessed through voltage maps with a common‐average reference. Among the 108 patients, 15 did not exhibit any IEDs during the single‐night assessment. The average number of IEDs per hour was expressed as the IEDs index (EDI), separately for NREM and REM sleep stages.

### 2.4 Statistical analysis

We employed generalized linear models to examine the association between the severity of OSA and EDI within specific sleep stages (NREM and REM). To better understand how IEDs are influenced by OSA severity, we applied internationally recognized AHI thresholds for our analysis (Berry et al., 2017). Specifically, we conducted a stepwise comparison using the following AHI thresholds: (1) AHI ≥ 5 (ranging from mild to severe OSA) versus AHI < 5 (no OSA); (2) AHI ≥ 15 versus AHI < 15; and (3) AHI ≥ 30 versus AHI < 30. The analysis was controlled for potential confounding factors, including patients' age, sex, type of epilepsy, use of antiepileptic medication (binary) and disease duration. Statistical analyses were carried out using SPSS for Windows (version 26.0, IBM), with significance determined at a two‐sided *p*‐value threshold of < 0.05.

## 3 RESULTS

In our study, 71% of patients had OSA, 58% were men and 64% had focal epilepsy. Additional cohort characteristics are detailed in Table 1. As indicated in Table 2, OSA severity was associated with indicators of impaired overall sleep, such as increased wake time after sleep onset. Moreover, the total number of IEDs during NREM sleep was higher compared with those recorded during REM sleep. More information on the overnight sleep study results and ED characteristics can be found in Table 2.

**TABLE 1:** Characteristics of patient cohort.

Characteristic	OSA severity			
	No OSA	Mild	Moderate	Severe
Patients with epilepsy, *n*	31	32	20	25
Age, mean (SD), years	43.0 (23.2)	52.5 (22.8)	58.1 (18.3)	74.0 (10.5)
Sex, *n* (%)				
Men	16 (51.6)	19 (59.4)	12 (60.0)	16 (64.0)
Women	15 (48.4)	13 (40.6)	8 (40.0)	9 (36.0)
Type of epilepsy, *n* (%)				
Focal epilepsy	12 (38.7)	26 (81.3)	14 (70.0)	17 (68.0)
Generalized epilepsy	19 (61.3)	6 (18.7)	6 (30.0)	8 (32.0)
Time elapsed since epilepsy diagnosis, *n* (%)				
≤ 1 year	15 (48.4)	20 (62.5)	8 (40.0)	17 (68.0)
≤ 10 years	12 (38.7)	7 (21.9)	11 (55.0)	5 (20.0)
> 10 years	4 (12.9)	5 (15.6)	1 (5.0)	3 (12.0)
No medication to treat epilepsy, *n* (%)	16 (51.6)	18 (56.3)	11 (55.0)	19 (76.0)
Use of antiepileptic drugs, *n* (%)				
Sodium valproate	4 (12.9)	7 (21.9)	6 (30.0)	1 (4.0)
Topiramate	2 (6.5)	1 (3.1)	0 (0.0)	1 (4.0)
Lamotrigine	4 (12.9)	1 (3.1)	0 (0.0)	0 (0.0)
Oxcarbazepine	7 (22.6)	4 (12.5)	1 (5.0)	1 (4.0)
Carbamazepine	1 (3.2)	2 (6.3)	1 (5.0)	2 (8.0)
Levetiracetam	1 (3.2)	1 (3.1)	0 (0.0)	1 (4.0)
Phenytoin sodium	0 (0.0)	0 (0.0)	1 (5.0)	0 (0.0)
Other medication used to treat epilepsy, *n* (%)				
Clonazepam	1 (3.2)	2 (6.3)	0 (0.0)	1 (4.0)

*Note*: That patients may have taken more than one medication to treat their epilepsy. OSA severity was determined by the AHI: no OSA = AHI < 5; mild OSA = AHI 5– < 15; moderate OSA = AHI 15– < 30; and severe OSA = AHI ≥ 30 events per hour.

Abbreviation: OSA, obstructive sleep apnea.

**TABLE 2:** Sleep characteristics among patients with epilepsy during a single‐night in‐hospital sleep study.

Sleep characteristic	OSA severity			
	No OSA, *N* = 31	Mild, *N* = 32	Moderate, *N* = 20	Severe, *N* = 25
Sleep‐onset latency, min	15.2 (13.6)	16.6 (17.8)	18.8 (29.0)	24.4 (27.9)
TST, min	451.4 (93.7)	412.7 (84.0)	407.7 (78.0)	361.9 (89.3)
Time in NREM sleep, min	381.7 (82.9)	349.8 (75.6)	340.5 (73.0)	316.7 (80.0)
Time in NREM sleep, %TST	84.6 (6.0)	84.9 (6.2)	83.4 (5.9)	87.7 (8.7)
Time in REM sleep, min	69.7 (32.5)	62.8 (29.6)	67.2 (26.3)	45.2 (40.8)
Time in REM sleep, %TST	15.4 (6.0)	15.1 (6.2)	16.6 (5.9)	12.3 (8.7)
Wake after sleep onset, min	78.2 (52.6)	112.0 (66.4)	84.3 (40.8)	167.4 (87.6)
Sleep efficiency, %	82.5 (10.1)	76.4 (11.1)	79.8 (9.2)	65.7 (14.5)
AHI, events per hr				
NREM sleep	2.3 (1.2)	7.8 (2.9)	20.4 (4.4)	50.1 (14.2)
REM sleep	3.8 (4.1)	16.7 (11.8)	27.4 (11.7)	38.5 (21.9)
IEDs, total number				
NREM sleep	3.9 (6.6)	3.8 (4.1)	4.3 (4.5)	7.0 (8.3)
REM sleep	1.2 (2.1)	3.0 (6.3)	1.5 (2.8)	1.5 (3.0)
EDI, IED events per hr				
NREM sleep	0.6 (0.8)	0.7 (0.7)	0.8 (0.9)	1.3 (1.6)
REM sleep	0.8 (1.2)	3.4 (7.7)	1.1 (1.6)	1.9 (3.5)

*Note*: Data are shown as mean (SD). OSA severity was determined by the AHI: no OSA = AHI < 5; mild OSA = AHI 5– < 15; moderate OSA = AHI 15– < 30; and severe OSA = AHI ≥ 30 events per hour.

Abbreviations: AHI, apnea–hypopnea index; EDI, interictal epileptiform discharges index; IEDs, interictal epileptiform discharges; NREM, non‐rapid eye movement; OSA, obstructive sleep apnea; REM, rapid eye movement; TST, total sleep time.

**FIGURE 1:**
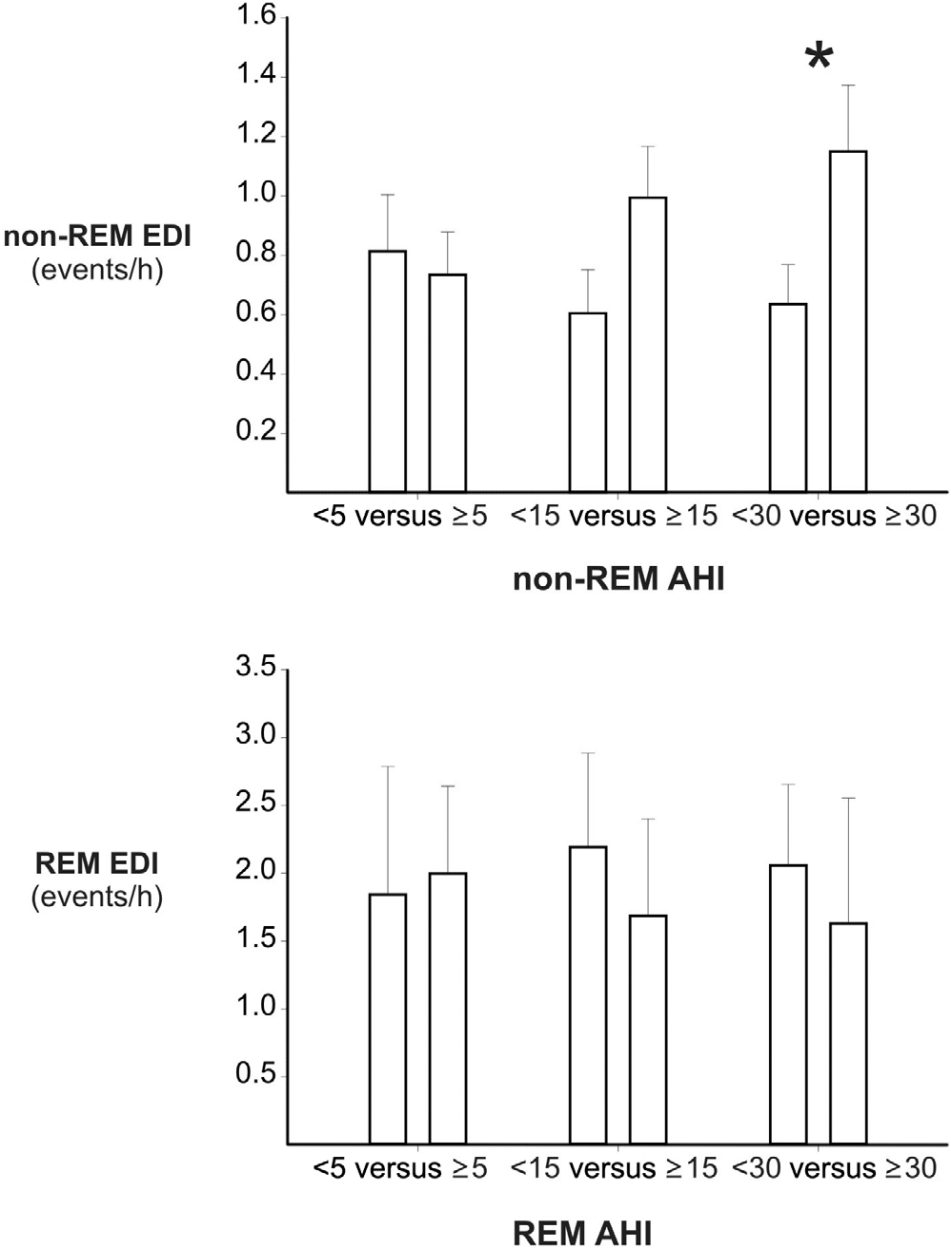
Threshold‐dependent association between non‐rapid eye movement (NREM) obstructive sleep apnea (OSA) and interictal epileptiform discharges (IEDs) in patients with epilepsy. The upper panel illustrates the threshold‐dependent relationship between OSA events, measured by the apnea–hypopnea index (AHI), and the hourly frequency of IEDs, expressed as the IEDs index (EDI), during NREM sleep. The lower panel shows the threshold‐dependent relationship between OSA events and the EDI during rapid eye movement (REM) sleep. *p < 0.05, as determined by generalized linear models adjusted for patients' age, sex, type of epilepsy, use of antiepileptic medication, and disease duration.

As illustrated in Figure 1, a threshold‐dependent association was found between OSA severity and EDI during NREM sleep. Specifically, patients with severe OSA exhibited a 0.51 (95% confidence interval [CI], −1.00, −0.02) higher NREM EDI compared with those with fewer OSA events (*p* = 0.04), as determined by adjusted generalized linear models. In contrast, no such significant threshold‐dependent associations were observed between OSA severity and the EDI during REM sleep (Figure 1).

To further investigate the relationship between NREM IEDs and a key characteristic of OSA, wake after sleep onset, we used generalized linear models. In these models, the NREM EDI served as the sole predictor, while the number of minutes awake between sleep onset and offset was the outcome. The analysis revealed a significant positive association between the frequency of NREM IEDs and the time spent awake (B = 13.9 min, SE = 6.4 min, *p* = 0.03). This indicates that for every additional NREM IED per hour, there is an average increase of 13.9 min in wakefulness after sleep onset.

## 4 DISCUSSION

In our single‐night video‐polysomnography study involving 108 patients with epilepsy, over 70% of whom had OSA, we identified a threshold‐dependent relationship between OSA severity and the presence of IEDs during NREM sleep. Specifically, patients with at least 30 apnea and hypopnea events per hour of NREM sleep exhibited a significantly higher hourl frequency of IEDs during this sleep stage compared with those with fewer OSA events. Additionally, patients with a higher frequency of NREM IEDs spent more time awake during the night, suggesting that IEDs during NREM sleep may contribute to sleep disruption in this population. In contrast, no threshold‐dependent association was observed between OSA severity and IEDs during REM sleep.

The question is how OSA events might trigger IEDs during NREM sleep? Respiration has been shown to influence brain oscillations (Kluger & Gross, 2021; Zelano et al., 2016), including brain activity during NREM sleep (Schreiner et al., 2023). Given this regulatory effect of breathing on brain activity, the recurrent episodes of partial or complete breathing cessation seen in severe OSA could potentially provoke IEDs in patients with epilepsy. However, this hypothesis requires further investigation.

Several limitations should be considered when interpreting our findings. First, 15 patients did not show any IEDs during the single‐night assessment, indicating a need for future research to examine the relationship between OSA and IEDs over multiple nights for a more comprehensive understanding. Multiple nights of assessment could also help mitigate first‐night effects, which might explain why 6% of the initially recruited 119 patients with epilepsy were excluded due to sleeping less than 4 hr. Another limitation is the high prevalence of OSA in our sample (~71%), likely because patients were referred to our clinic with suspected comorbid conditions such as OSA. This high prevalence may limit the generalizability of our findings to the broader population of Asian patients with epilepsy. Additionally, the low prevalence of IEDs observed may be partly due to our rigorous methodology, which required consensus for IED identification. While this approach minimized individual biases and increased precision, it may have led to a more conservative classification, potentially excluding borderline cases and underestimating the prevalence of IEDs in our sample.

## 5 CONCLUSIONS

Our findings highlight that severe OSA is linked to an increased frequency of IEDs during NREM sleep, which, in turn, is associated with greater time awake between sleep onset and offset. Further studies are warranted to validate our findings across diverse ethnicities, and over multiple nights of sleep and IED recordings.

## AUTHOR CONTRIBUTIONS

**Meina Wu:** Conceptualization; writing – original draft; methodology; visualization; formal analysis; data curation. **Pei Xue:** Writing – review and editing; formal analysis; visualization; conceptualization. **Jinzhu Yan:** Funding acquisition; writing – review and editing; methodology; project administration; supervision; data curation; conceptualization. **Christian Benedict:** Writing – review and editing; supervision; conceptualization.

## FUNDING INFORMATION

Funding/support for this research was provided by the Natural Science Foundation of Fujian Province, China (grant number 2019J01095), with support from Yan. Additionally, C.B. declares that their work is supported by the Swedish Brain Foundation, although this specific study did not receive funding from them. The funder had no role in the design and conduct of the study; the collection, management, analysis or interpretation of the data; the preparation, review or approval of the manuscript; and the decision to submit the manuscript for publication.

## CONFLICT OF INTEREST STATEMENT

The authors declare that they have no conflicts of interest related to this research.

## Data Availability

The data are available upon request to researchers from other universities, who are required to sign a data access agreement prior to release. For inquiries regarding access to the data, please contact meinawu@163.com (Meina Wu).
